# Prescription of lipid-lowering medications for patients with type 2 diabetes mellitus and risk-associated LDL cholesterol: a nationwide study of guideline adherence from the Swedish National Diabetes Register

**DOI:** 10.1186/s12913-018-3707-4

**Published:** 2018-11-28

**Authors:** Sofia Axia Karlsson, Stefan Franzén, Ann-Marie Svensson, Mervete Miftaraj, Björn Eliasson, Karolina Andersson Sundell

**Affiliations:** 1 Department of Public Health and Community Medicine, Sahlgrenska Academy, University of Gothenburg, BOX 453, SE-405 30 Gothenburg, Sweden; 2 National Diabetes Register, Centre of Registers, Gothenburg, Sweden; 3 Department of Molecular and Clinical Medicine, Sahlgrenska Academy, University of Gothenburg, Gothenburg, Sweden; 4 AstraZeneca AB, Medical Evidence and Observational Research, Mölndal, Sweden

**Keywords:** Type 2 diabetes mellitus, Diabetes care, Guideline adherence, Lipid-lowering medications, Cardiovascular disease prevention

## Abstract

**Background:**

Management of type 2 diabetes mellitus (T2DM) encompasses intensive glycaemic control, along with treatment of comorbidities and complications to handle the increased risk of cardiovascular disease (CVD). Improved control of LDL-cholesterol (LDL-C) with lipid-lowering medications is associated with reduced CVD risk in T2DM patients. Thus, treatment guidelines recommend lipid-lowering medications for T2DM patients with LDL-C above risk-associated thresholds. This study aimed to assess healthcare provider adherence to guidelines regarding lipid-lowering medication prescription among T2DM patients and to analyse factors associated with lipid-lowering medication prescription.

**Methods:**

Observations in 2007 − 2014 for T2DM patients age ≥ 18 were collected from the Swedish National Diabetes Register. Observations were excluded if they lacked information about LDL-C, lipid-lowering medication prescription or CVD. Observations with established CVD were attributed to secondary prevention; remaining observations were attributed to primary prevention. The analyses included primary and secondary prevention observations with LDL-C above risk-associated thresholds (LDL-C ≥ 2.5 mmol/l and LDL-C ≥ 1.8 mmol/l respectively). Guideline adherence was analysed as the probability of prescribing lipid-lowering medications using mixed-effect model regression adjusted for potential confounders. Factors associated with prescribing lipid-lowering medications were analysed for patient and healthcare provider characteristics using mixed-effect model regression and odds ratio.

**Results:**

A total of 1,204,376 observations from 322,046 patients reported by 1352 healthcare providers were included. Primary prevention accounted for 63%; 52% were men, mean age was 64 and mean LDL-C was 3.4 mmol/l. For secondary prevention, 60% were men, mean age was 72 and mean LDL-C was 2.7 mmol/l. During 2007–2014, guideline adherence ranged from 36 to 47% for primary prevention and 59 to 69% for secondary prevention. In general, concomitant prescription of diabetes medications, antiplatelets and antihypertensives along with smoking and specialised care were associated with higher prescription of lipid-lowering medications. Patients age ≥ 80 were associated with lower prescription of lipid-lowering medications. Higher prescription was associated with longer diabetes duration in primary prevention and men in secondary prevention.

**Conclusions:**

Adherence to treatment guidelines levelled off after an initial increase in both prevention groups. Lipid-lowering medication prescription was based on individualised CVD risk.

**Electronic supplementary material:**

The online version of this article (10.1186/s12913-018-3707-4) contains supplementary material, which is available to authorized users.

## Background

Management of type 2 diabetes mellitus (T2DM) includes intensive glycaemic control, along with treatment of comorbidities and complications to combat the increased risk of cardiovascular disease (CVD) [1–3]. Thus, risk factor control is an important part of treatment guidelines in order to prevent CVD. Treatment guidelines are regularly updated based on new evidence and as new treatment regimens becomes available. Many guidelines focus on threshold values, yet in recent years the approach has shifted to focus on patient’s risk profile using tools such as risk engines. Improved control of low-density lipoprotein cholesterol (LDL-C) with lipid-lowering medications is associated with reduced risk of CVD and mortality in patients with T2DM [4–6]. Thus, international treatment guidelines recommend lipid-lowering medications for T2DM patients with an LDL-C above risk-associated thresholds until 2015 [7–10]. Before 2015, the Swedish national treatment guidelines for diabetes care recommended prescription of lipid-lowering medications for all patients with T2DM and LDL-C ≥ 2.5 mmol/l [11]. But for T2DM patients with established CVD, the European Society of Cardiology and later the International Diabetes Federation and the American Diabetes Association recommended lipid-lowering medications to reduce LDL-C below 1.8 mmol/l [1, 3, 12].

Treatment guidelines are based on outcomes from large randomised controlled studies and are intended for patients without conditions contraindicated for medication use. Adherence [13] to such guidelines could improve the quality of clinical decisions and help identify areas in need of further development to ensure optimal care [14]. Non-adherence to treatment guidelines includes healthcare providers who fail to initiate or intensify therapy, which may result in unsatisfactory medical outcomes [15]. Guideline adherence does not represent patient adherence as not all prescriptions are filled. Thus, these terms should not be confused with each other.

Previous studies have reported guideline adherence among patients with diabetes mellitus as varying between 24 and 80%, finding that T2DM patients and/or secondary prevention patients are more likely to receive lipid-lowering medications [16–21]. Furthermore, concomitant prescription of other cardioprotective medications is associated with higher prescription of lipid-lowering medications [16]. Age above 75 [17, 19] and low kidney function [22] are considered contraindications and have been shown to be associated with lower odds of receiving lipid-lowering medications. Few studies have assessed guideline adherence to lipid-lowering medications in both primary and secondary prevention.

Knowledge about adherence to Swedish treatment guidelines for diabetes care is scarce. Thus, the aim of this study, using nationwide data from primary and specialised care, was to assess Swedish healthcare providers’ guideline adherence to prescription of lipid-lowering medications among T2DM patients for primary and secondary prevention of CVD between 2007 and 2014, as well as to identify factors associated with lipid-lowering medication prescription.

## Methods

### Data sources

In this population-based study, patient data were collected from the Swedish National Diabetes Register (NDR), which is estimated to include 90% of all Swedish patients with T2DM age 18 or older [23]. Since its launch in 1996, the register has been trying to improve the quality of diabetes care, offering up-to-date information about diabetes treatment, clinical characteristics and presence of complications in order to promote evidence-based trends [23]. Patient data are continuously reported by physicians and nurses at primary and secondary care clinics nationwide, through either electronic medical records or the NDR website. Reported data is collected at healthcare facility levels, thus not on individual practitioner level. In Sweden, one-person healthcare practices are uncommon. All patients provided informed consent for inclusion in the NDR and thus inclusion in the present study.

### Study population

All observations for patients with T2DM age 18 or older entered in the NDR between 1 January 2007 and 31 December 2014 (the study period) were eligible for this study. Patients included in the study had at least one entry in the NDR. Observations were included if they had available information about LDL-C, prescription of lipid-lowering medications and CVD. Hence, observations were excluded if they lacked information on either LDL-C, prescription of lipid-lowering medications or CVD. Each patient could contribute with several observations during the study period. However, longitudinal assessment of within-patient patterns was not performed. Observations with established CVD were attributed to secondary prevention; all other observations were attributed to primary prevention. For patients with observations that lacked information about CVD, all observations preceding the latest one without CVD were assigned to no established CVD, and all observations subsequent to the first observation with CVD were assigned to established CVD. Observations for primary prevention with LDL-C < 2.5 mmol/l and secondary prevention with LDL-C < 1.8 mmol/l were considered to be below the risk-associated thresholds and thus excluded from the analyses.

### Outcomes

The primary outcome was healthcare providers’ adherence to guidelines for prescribing lipid-lowering medications among patients with T2DM. The secondary outcome included factors associated with prescription of lipid-lowering medications. In the NDR, prescription of lipid-lowering medications is categorised as yes or no. Prevalence of lipid-lowering medication prescription was assessed for the study population and guideline adherence was measured as the probability of prescribing lipid-lowering medications for T2DM patients with LDL-C ≥ 2.5 mmol/l in primary prevention and/or ≥ 1.8 mmol/l in secondary prevention, adjusted for potential confounders. The odds of prescribing lipid-lowering medications were assessed for patient and healthcare provider characteristics.

### Covariates

Patient characteristics included sex, age, clinical variables (diabetes duration, haemoglobin A1c [HbA1c], estimated glomerular filtration rate [eGFR], blood pressure, blood lipid levels, body mass index [BMI], microalbuminuria, macroalbuminuria, physical activity, smoking) and prescriptions (diabetes medications, antiplatelets, antihypertensives).

Sex, microalbuminuria, macroalbuminuria, smoking and prescriptions were categorised dichotomously. Smoking was defined as at least one cigarette per day, pipe or cessation within the past 3 months. Physical activity was defined as a 30-min walk or the equivalent and categorized as less than once/week, 1–2 times/week, 3–5 times/week or daily. Age was categorised as ≤ 60, 61–80 and > 80, and diabetes duration as < 5, 5–10 and ≥ 10 years. BMI was categorized as < 30 and ≥ 30 kg/m^2^. The following variables were categorised as high or low according to cut-offs that represented the recommended target values at the time of the study [11]: HbA1c, 52 mmol/mol; eGFR, 60 ml/min/1.73 m2; systolic blood pressure, 130 mmHg; diastolic blood pressure, 80 mmHg; total cholesterol, 4.5 mmol/l; HDL cholesterol, 1.0/1.3 (men/women) mmol/l; triglycerides, 2.0 mmol/l. LDL-C was used as a continuous variable.

Healthcare providers were identified at healthcare centre level and were characterised according to county council and type of care (primary or specialised care).

### Statistical analysis

The prevalence of lipid-lowering medication prescription was assessed and compared between observations independent of LDL-C values and those with LDL-C above the risk-associated thresholds. The probability of prescribing lipid-lowering medications according to guidelines was analysed by year using mixed model regression adjusted for potential confounders. To identify factors associated with prescription of lipid-lowering medications, the probability of prescription was analysed for all patient and healthcare provider characteristics using mixed model regression and odds ratios. In the mixed model regression, lipid-lowering medication prescription was considered the response, patient and healthcare provider characteristics were considered random effects and patients’ unique identifier were considered repeated fixed effects.

To analyse the impact of conditions contraindicated for lipid-lowering medications, the probability of adherence to guidelines was assessed and compared between the study population and exclusion of T2DM patients age 80 or older and/or with eGFR< 30 ml/min/1.73 m2.

The analyses were performed separately for observations attributed to primary and secondary prevention. All statistical analyses were performed using SAS Software 9.4 (SAS institute, Cary NC).

### Ethics approval

The study was approved by the Regional Ethical Review Board at the University of Gothenburg (Reg. no. 1173-16). Before reporting to the NDR, all patients were informed (verbally or in writing) about the register and the possibility to decline or being excluded from the register. Hence, individual consent was not required for the present study.

## Results

### Study population

**Fig. 1:**
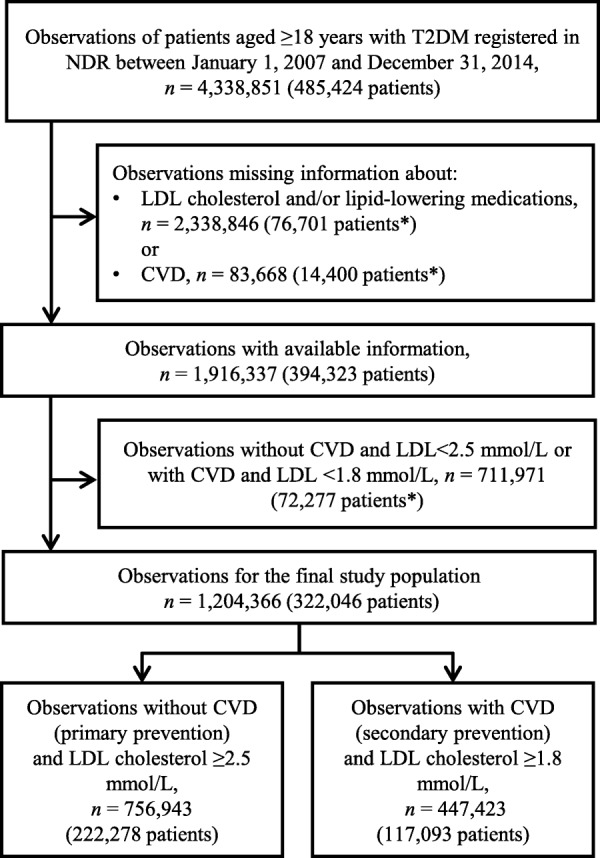
Exclusion criteria for the study population. An asterisk (*) indicates the number of patients who were completely excluded due to current exclusion criteria

A total of 1,204,376 observations among 322,046 T2DM patients reported by 1352 healthcare providers (93.1% primary healthcare centres) were included (Fig. 1). A total of 64% of all patients were attributed to primary prevention only and 31% to secondary prevention only, while 5% experienced their first CVD during the study period and were thus attributed to both primary and secondary prevention.

For primary prevention, 52% were men, the mean age was 64 and the mean LDL-C was 3.4 mmol/l (Table 1). Prescription of antihypertensives and antiplatelets was found in 70 and 23% of the observations respectively. For secondary prevention, 60% were men, the mean age was 72 and the mean LDL-C was 2.7 mmol/l. Prescription of antihypertensives and antiplatelets was found in 90 and 70% of the observations respectively. For both prevention groups, the mean HbA1c was above 52 mmol/mol and diabetes medications had been prescribed in more than 70% of the observations. Patients characteristics were similar over time, with the exception of prescription of antiplatelets, which decreased for both prevention groups (Additional files 1 and 2).

**Table 1:** Characteristics of the study population

Characteristics	Primary prevention *n* = 756,953	Secondary prevention *n* = 447,423
Men, n (%)	396,028 (52.3)	274,891 (61.4)
Age, mean	63.7 ± 11.7	71.5 ± 9.5
Diabetes duration, mean years	7.4 ± 7.1	10.5 ± 8.4
HbA1c, mean mmol/mol	54.7 ± 14.6	55.5 ± 13.7
Diabetes medications, n (%)	560,539 (74.4)	350,387 (78.5)
Antiplatelets^a^, n (%)	171,114 (23.1)	316,290 (72.1)
Antihypertensives, n (%)	508,907 (68.6)	401,935 (90.9)
Systolic blood pressure, mean mmHg	137.2 ± 16.4	136.2 ± 16.9
Diastolic blood pressure, mean mmHg	78.5 ± 9.6	74.9 ± 9.8
Total cholesterol, mean mmol/l	5.4 ± 0.9	4.7 ± 1.0
LDL cholesterol, mean mmol/l	3.4 ± 0.7	2.7 ± 0.8
HDL cholesterol, mean mmol/l	1.3 ± 0.4	1.2 ± 0.3
Triglycerides, mean mmol/l	1.8 ± 0.9	1.8 ± 0.9
eGFR, mean ml/min/1.73 m²	85.6 ± 25.1	74.3 ± 25.0
Microalbuminuria, n (%)	92,812 (17.8)	77,698 (25.6)
Macroalbuminuria, n (%)	29,895 (5.7)	35,005 (11.3)
BMI, mean kg/m^2^	30.1 ± 5.4	29.7 ± 5.1
Physical activity < once a week^b^, n (%)	105,300 (24.2)	87,486 (32.8)
Smoking^c^, n (%)	103,874 (17.1)	49,766 (13.7)

*Abbreviations*: HbA1c, haemoglobin A1c; LDL, low-density lipoprotein; HDL, high-density lipoprotein; eGFR, estimated glomerular filtration rate; BMI, body mass index

^a^ATC code B01AC and N02BA01

^b^30-minute walk or equivalent

^c^smoking at least one cigarette or pipe per day, or quit smoking within three months

### Prescription of lipid-lowering medications

**Fig. 2:**
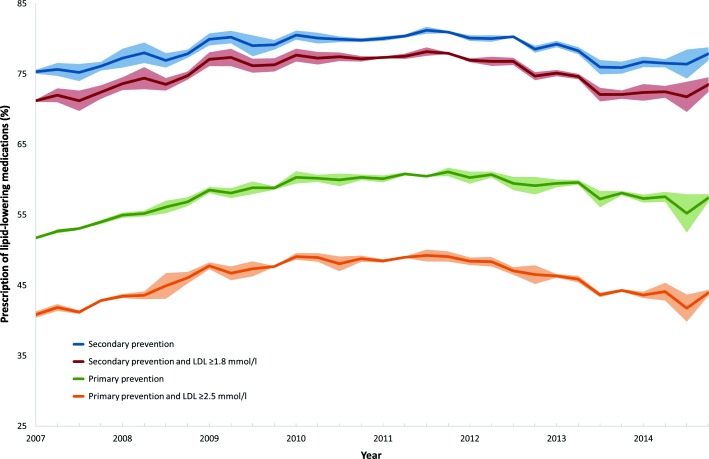
Proportion of observations where lipid-lowering medications had been prescribed in 2007–2014

Between 2007 and 2014, the prevalence of lipid-lowering medication prescription for observations with risk-associated LDL-C levels ranged from 40 to 49% in primary prevention and 72 to 78% in secondary prevention (Fig. 2). For primary prevention, the adjusted probability of prescribing lipid-lowering medications, accounting for potential confounders, and thus healthcare providers adherence to guidelines increased from 36% in 2007 to 47% in 2014 (Table 2). Corresponding guideline adherence for secondary prevention increased from 59 to 69%. For both prevention groups, according to visual inspection, the adjusted probability increased during the first 3 years and levelled off afterwards. No formal statistical testing was conducted on differences in the probability over time.

**Table 2:** Guideline adherence to, measured as the probability of, prescription of lipid-lowering medications by year

Year	Primary prevention		Secondary prevention	
	Crude (%) *n* = 756,943	Adjusted^a^ (%) *n* = 238,895	Crude (%) *n* = 447,423	Adjusted^a^ (%) *n* = 142,739
	Mean (95% CI)	Mean (95% CI)	Mean (95% CI)	Mean (95% CI)
2007	34.7 (34.3–35.2)	36.2 (34.8–37.5)	70.7 (70.2–71.2)	58.6 (56.8–60.5)
2008	39.0 (38.6–39.4)	40.3 (39.0–41.6)	72.9 (72.4–73.3)	63.0 (61.3–64.7)
2009	42.7 (42.4–43.1)	43.8 (42.4–45.1)	74.7 (74.3–75.1)	66.2 (64.5–67.8)
2010	44.8 (44.5–45.1)	46.3 (44.9–47.6)	74.7 (74.4–75.1)	67.8 (66.2–69.4)
2011	46.8 (46.5–47.1)	46.6 (45.2–48.0)	75.1 (74.8–75.4)	69.1 (67.5–70.7)
2012	47.4 (47.1–47.7)	46.6 (45.2–48.0)	73.6 (73.2–73.9)	67.9 (66.2–69.5)
2013	47.6 (47.3–47.9)	46.6 (45.2–48.0)	71.8 (71.5–72.2)	66.8 (65.1–68.4)
2014	48.3 (48.0–48.6)	46.1 (44.7–47.5)	70.9 (70.5–71.3)	65.9 (64.2–67.5)

^a^Adjusted for year, county council, type of care, sex, age, HbA1c, eGFR, diabetes duration, diabetes medications, antihypertensives, antiplatelets, blood pressure, microalbuminuria, macroalbuminuria, BMI, physical activity, smoking and cholesterol levels

**Fig. 3:**
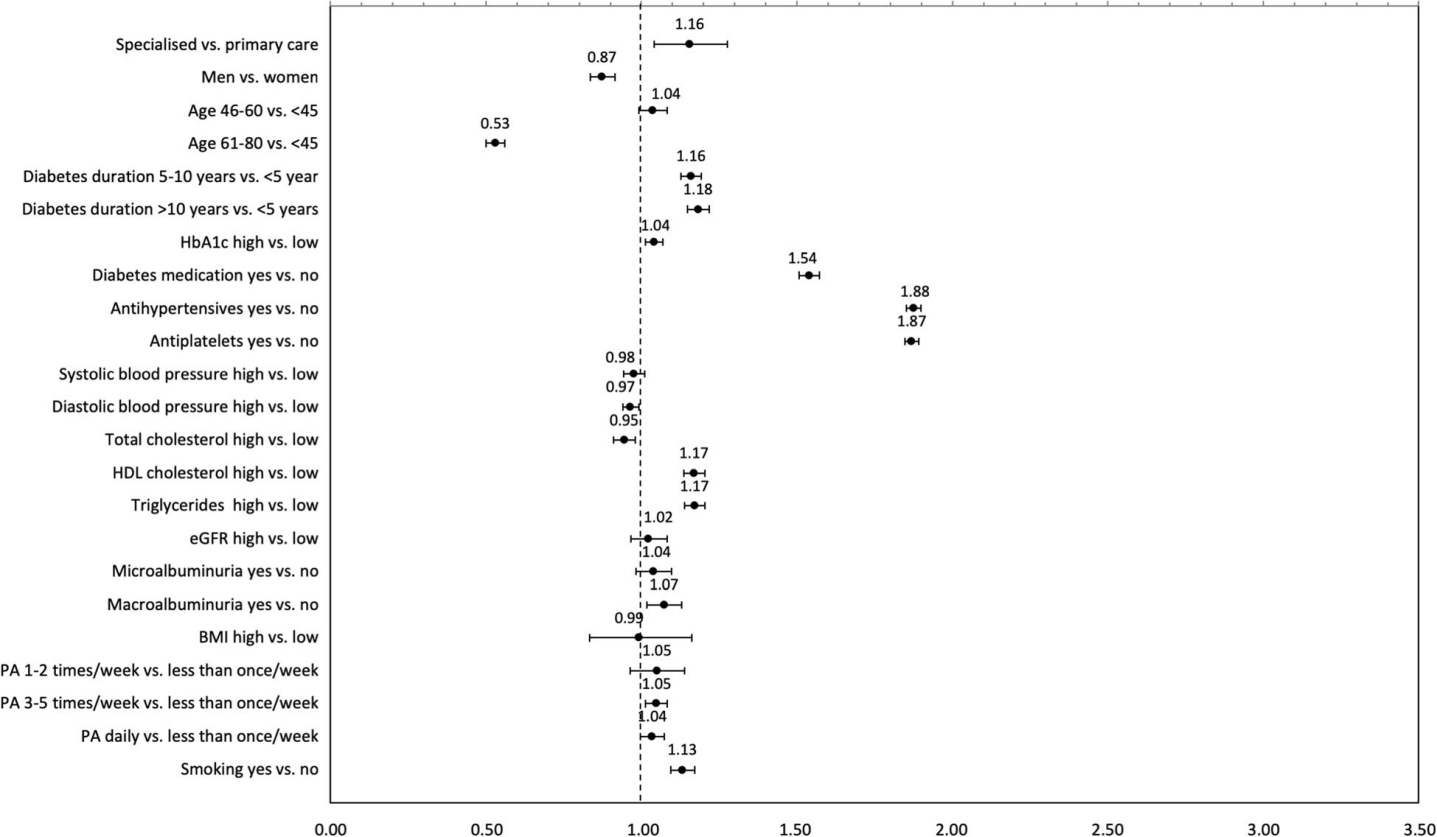
Odds ratio (95% CI) for patient and provider characteristics in observations attributed to primary prevention. Abbreviations: HbA1c, haemoglobin A1c; eGFR, estimated glomerular filtration rate; PA, physical activity

**Fig. 4:**
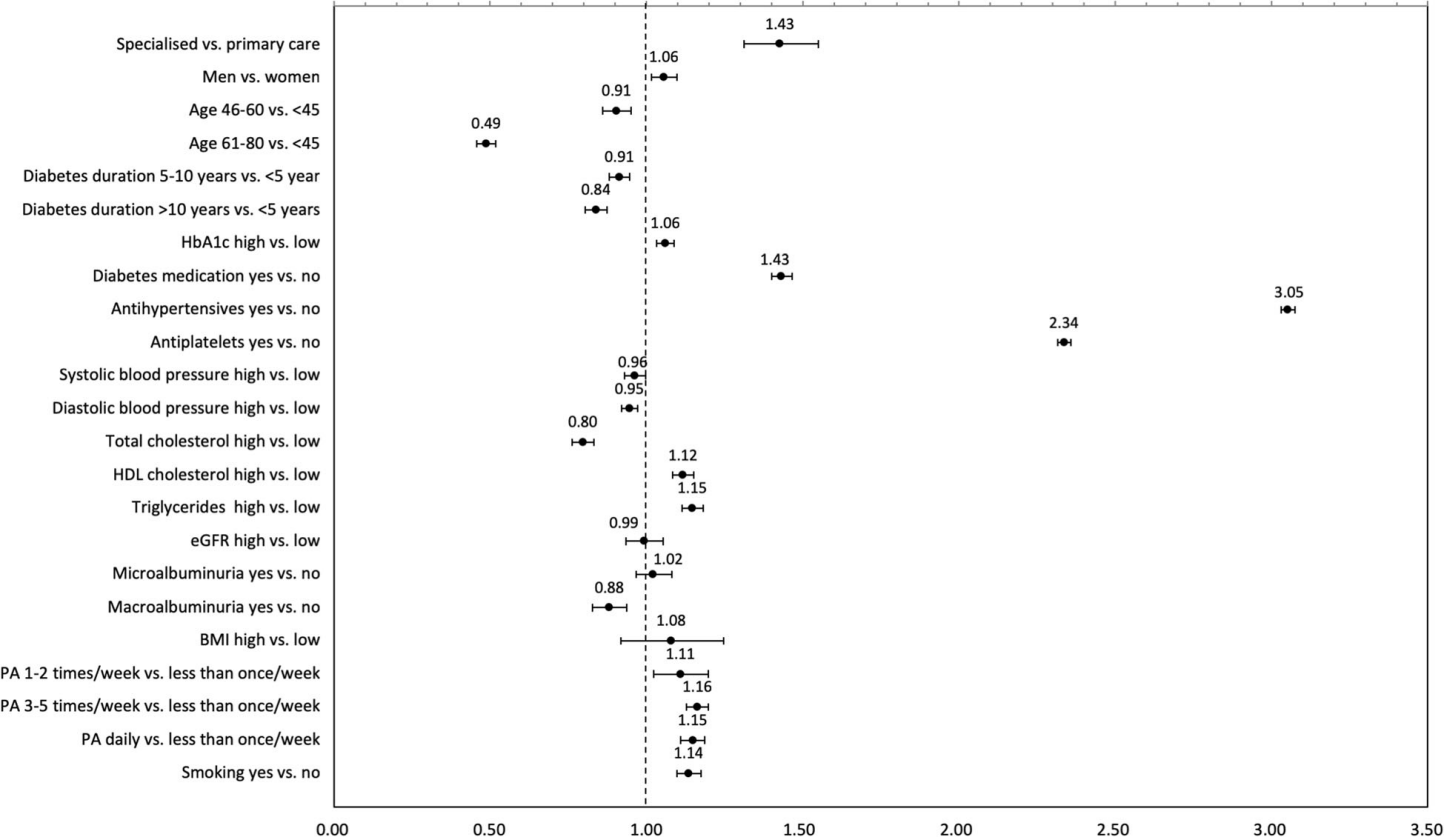
Odds ratio (95% CI) for patient and provider characteristics in observations attributed to secondary prevention. Abbreviations: HbA1c, haemoglobin A1c; eGFR, estimated glomerular filtration rate; PA, physical activity

Specialised healthcare providers were more likely to prescribe lipid-lowering medications for both prevention groups (OR = 1.16, 1.43) (Figs. 3 and 4). Patients who were physically active more than once a week had a higher chance of receiving lipid-lowering medication prescription (OR = 1.04–1.05 in primary prevention, OR = 1.11–1.16 in secondary prevention) as well as smokers (OR = 1.13, 1.14). The odds of receiving lipid-lowering medication prescription were lower among patients with high total cholesterol (OR = 0.88, 0.95) and higher among patients with high HbA1c (OR = 1.04, 1.06), high HDL cholesterol (OR = 1.12, 1.17) and high triglycerides (OR = 1.15, 1.17). For primary prevention, the odds of receiving lipid-lowering medication prescription were lower for men (OR = 0.88) and patients age 80 or older (OR = 0.53) (Fig. 3). The chance was higher with increasing diabetes duration (OR = 1.16, 1.19) and with concomitant prescription of diabetes medications (OR = 1.54), antihypertensives (OR = 1.87) or antiplatelets (OR = 1.87) as well as in presence of macroalbuminuria (OR = 1.07). For secondary prevention, the odds of receiving lipid-lowering medication prescription were lower with increasing age (OR = 0.89, 0.48) and presence of macroalbuminuria (OR = 0.87) (Fig. 4). A greater odds ratio for receiving lipid-lowering medication prescription was higher with concomitant prescription of diabetes medications (OR = 1.44), antihypertensives (OR = 3.06) or antiplatelets (OR = 2.33). No statistically significant difference was observed among remaining risk factors. Guideline adherence for lipid-lowering medications increased a few percentage points when excluding patients age ≥ 80 or older and/or eGFR < 30 ml/min/1.73m^2^ (Additional files 3, 4 and 5).

A total of 11.2% of information about patient characteristics was missing: 1.4% about prescribed medications and 15.1% about clinical variables. No information was missing about age, sex or healthcare provider characteristics.

## Discussion

In this nationwide study of more than 300,000 T2DM patients, prescription of lipid-lowering medications was found in half of the observations attributed to primary prevention and three-quarters of the observations attributed to secondary prevention in 2007-2014. When adjusted for potential confounders, the probability of prescribing lipid-lowering medications according to guidelines ranged from 36 to 47% in primary prevention and 59 to 69% in secondary prevention. We found different clinical factors to be associated with lipid-lowering medication prescription, which indicates that prescription was based on an individualised CVD risk assessment taking the risk-associated thresholds into account.

Our findings concur with previous research that reports varying guideline adherence to prescription of lipid-lowering medications between T2DM patients attributed to primary and secondary prevention, as well as the association between prescription and CVD risk factors [16, 17]. A previous study from the NDR showed that lipid-lowering medications were prescribed for 70% of T2DM patients with triglycerides > 4 mmol/l [18]. In the UK, lipid-lowering medications for primary prevention were prescribed for 48% of eligible patients with diabetes mellitus age 30-74 [16] and in 68% of T2DM patients age 40 or older in Scotland [17]. Among T2DM patients in Germany, lipid-lowering medications were prescribed for 24% in primary prevention, 46% in secondary prevention and 32% with prevention status unknown [19]. A total of 64% of T2DM patients treated in primary care were prescribed lipid-lowering medications in Australia [20]. Among U.S. veterans with diabetes mellitus (96% men) age 40-75, lipid-lowering medications were prescribed in 61% of primary prevention patients and in 80% of secondary prevention patients [21].

Since all included observations were considered eligible for lipid-lowering medications, the actual pattern suggests that healthcare providers based their prescription on more than LDL-C. The analyses showed that certain clinical factors were associated with higher odds of prescribing lipid-lowering medications. Some of these associations were common to primary and secondary prevention, whereas others varied between the prevention groups. Prescription of antihypertensives, antiplatelets and diabetes medications were all associated with higher odds of receiving lipid-lowering medication prescription in both primary and secondary prevention. This finding is in line with previous studies, which have shown that prescription of lipid-lowering medications is associated with concomitant prescription of cardioprotective medications for patients with diabetes mellitus [16]. Observations recorded by specialised healthcare providers were associated with higher odds as well. This association may be due to the fact that patients with more severe diabetes accompanied by serious comorbidities and complications had greater odds of being treated with several different medications and to receive specialised care. We found that primary and secondary prevention patients who smoked had higher odds of receiving lipid-lowering medications than non-smokers, which also concurs with previous studies [17].

When excluding patients age 80 or older and/or with low kidney function, the probability of prescribing lipid-lowering medications according to guidelines increased by a few percentage points in both primary and secondary prevention. This finding suggests that lipid-lowering medications were less likely to be prescribed for these patients. Previous studies have also reported lower prescription of lipid-lowering medications for T2DM patients age 75 or older [17, 19] or with lower kidney function [22]. Although elderly patients have a significantly higher risk of CVD, they are less likely to receive lipid-lowering medications [24, 25]. Ageing is often accompanied by comorbidities and changes in both pharmacodynamics and pharmacokinetics, which increases the risk of drug-related disease and drug-drug interactions [26]. However, lipid-lowering medications have been reported to be associated with lower risk of cardiovascular mortality in T2DM patients age 80 or older [24] and lower risk of CVD in T2DM patients with kidney disease not yet requiring renal replacement therapy [27].

Altogether, our findings suggest that Swedish healthcare providers based their lipid-lowering medication prescription for T2DM patients on the individual’s risk of developing CVD, rather than LDL-C values alone. Previously, the clinical value of LDL-C as a risk marker for CVD has been discussed and the ratio of non-HDL to HDL cholesterol has been shown to be a better measure for patients with diabetes mellitus [28, 29]. In 2015, Swedish treatment guidelines for diabetes care were revised to recommend that the decision to prescribe lipid-lowering medications should be based on the individual’s estimated 10-year risk of fatal CVD, using SCORE charts [30, 31]. This risk assessment is based on several risk factors, including sex, age, cholesterol level, blood pressure and smoking status. In 2012, the NDR adopted a model that estimates the patient’s 5-year risk of a fatal or non-fatal cardiovascular event in a Swedish setting [32]. This approach is considered to be more accurate than the 10-year risk estimate. The model is available on the NDR website [33] as a tool to facilitate and support clinical decisions, such as prescription of medications.

Treatment guidelines will never be fully applied in clinical practice. When a patient is diagnosed with T2DM, the guidelines recommend initiation of an extensive treatment regimen that includes several different medication classes [3, 11]. Initiation of medications should be a joint decision of the healthcare provider and patient. Thus, prescription of lipid-lowering medications may be considered a later stage in the implementation of diabetes treatment. For patients who experience a cardiovascular event, however, lipid-lowering medications have higher priority and are recommended in close conjunction with the event. Reasons for not prescribing lipid-lowering medications include previous adverse effects from such medications, refused treatment or preference for behavioural changes first [34]. Statins are the most commonly prescribed lipid-lowering medications and have been shown to be cost-effective for both primary [35] and secondary [36, 37] prevention of CVD among T2DM patients. Lipid-lowering medications (including statins) for prevention of CVD are included in the Swedish reimbursement system and patients pay a low deductible with each filled prescription. Thus, it is unlikely that public and patient costs are a disincentive for prescription.

Guideline adherence should not be confused with actual patient adherence. However, prescription is a prerequisite for patients to collect their medications at a pharmacy. Healthcare providers’ attitudes and beliefs concerning management of diabetes have been shown to influence self-management behaviour among T2DM patients [15]. Patient perceptions of diabetes are influenced by the healthcare professionals they encounter. Clear communication between patient and provider is a predictor of good self-management, whereas poor communication is associated with poor treatment adherence. A recent Swedish study reported that improved control of HbA1c among T2DM patients was associated with human resources and organisational features of primary healthcare clinics [38], including access to a diabetes team, group training programmes and registered nurses with diabetes education. This finding suggests that the characteristics of healthcare providers who treat T2DM patients also play a major role in initiation and implementation of treatment.

### Strengths and limitations

The strengths of the study include the large population of more than 300,000 T2DM patients and extensive adjustments for clinical risk factors at both primary and specialised care clinics. The comprehensive material obtained from the NDR allowed us to assess guideline adherence over time and among T2DM patients attributed to primary and secondary prevention of CVD. To the best of our knowledge, few studies have included both primary and secondary prevention patients. Thus, this study contributes with information that confirms the results of previous studies and provides additional knowledge about differences in the prescription of lipid-lowering medications between primary and secondary prevention patients with T2DM.

Data reflect what which has been entered as prescribed by healthcare providers, so we cannot be sure of whether lipid-lowering medication prescriptions were issued or not. No actual information about prescription is registered. However, entered data are often transferred directly from medical records such that prescription is entered only if the medication has been prescribed. Since not all variables were measured at each medical visit, presence of missing data is substantial. Lack of information about LDL-C and/or prescription of lipid-lowering medications comprised the majority of excluded observations due to missing information. Assessment of patient characteristics between observations with and within missing information showed most similarities between the groups, although some differences was observed. Hence, we cannot rule out that missing data occurred not at random, which may have introduced bias to the study.

Another limitation is the lack of information about risks for and presence of drug-related disease or drug-drug interactions, which are not included in the NDR (such as myopathy, Parkinson’s disease, liver function, liver failure, drug interactions, etc.). Thus, we may have underestimated guideline adherence among T2DM patients by including those with possible contraindications for lipid-lowering medications.

## Conclusions

From 2007 to 2014, Swedish healthcare providers’ prescription of, and thus their guideline adherence to, lipid-lowering medications increased during the first years and thereafter levelled off for primary prevention and fluctuated year-by-year for secondary prevention. Adherence to guidelines was associated with different CVD risk factors among patients attributed to primary and secondary prevention, respectively. This information is valuable for guideline and policymakers, as well as healthcare providers who treat patients with T2DM, to evaluate the healthcare system in order to ensure optimal, standardised and equal care.

## Additional files

Additional file 1: Patient characteristics by year for observations attributed to primary prevention. (PDF 29 kb)

Additional file 2: Patient characteristics by year for observations attributed to secondary prevention. (PDF 29 kb)

Additional file 3: Probability of prescribing lipid-lowering medications by year for patients younger than 80 and eGFR > 30 ml/min/1.73 m^2^. (PDF 61 kb)

Additional file 4: Probability of prescribing lipid-lowering medications by year for patients younger than 80. (PDF 61 kb)

Additional file 5: Probability of prescribing lipid-lowering medications by year for patients with eGFR > 30 ml/min/1.73 m^2^. (PDF 61 kb)

## Abbreviations

BMI: Body mass index
CVD: Cardiovascular disease
eGFR: Estimated glomerular filtration rate
HbA1c: Haemoglobin A1c
NDR: Swedish National Diabetes Register
T2DM: Type 2 diabetes mellitus
