# Supplementary material for: Prescription of lipid-lowering medications for patients with type 2 diabetes mellitus and risk-associated LDL cholesterol: a nationwide study of guideline adherence from the Swedish National Diabetes Register
Source: BMC Health Serv Res. 2018 Nov 28;18:900. doi: 10.1186/s12913-018-3707-4 (PMC6260691; doi:10.1186/s12913-018-3707-4)
Supplement: Supplementary file 1 — Patient characteristics by year for observations attributed to primary prevention. (PDF 29 kb) [file 12913_2018_3707_MOESM1_ESM.pdf]

	2007	2008	2009	2010	2011	2012	2013	2014
Characteristics	n=45,091	n=55,452	n=68,892	n=106,258	n=132,783	n=128,542	n=108,874	n=111,051
Men, n (%)	23,642 (52.4)	28,974 (52.3)	36,324 (52.7)	55,202 (52.0)	69,039 (52.0)	67,506 (52.5)	56,896 (52.3)	58,445 (52.6)
Age, mean	63.6 ± 11.4	63.6 ± 11.4	63.5 ± 11.5	63.5 ± 11.6	63.6 ± 11.7	63.8 ± 11.7	63.9 ± 11.9	64.1 ± 12.0
Diabetes duration, mean years	7.7 ± 7.1	7.5 ± 7.0	7.5 ± 7.1	7.2 ± 6.9	7.2 ± 7.0	7.3 ± 7.0	7.4 ± 7.1	7.6 ± 7.2
HbA1c, mean mmol/mol	53.5 ± 13.2	53.9 ± 13.2	54.1 ± 13.5	54.7 ± 13.8	54.7 ± 14.5	55.4 ± 15.1	54.8 ± 15.5	54.9 ± 15.8
Diabetes medications, n (%)	32,869 (73.1)	39,809 (71.9)	49,632 (72.2)	78,541 (74.0)	98,594 (74.4)	96,596 (76.1)	81,390 (74.9)	83,108 (75.2)
Antiplatelets[c], n (%)	13,774 (31.2)	16,102 (29.9)	19,027 (28.5)	27,334 (26.2)	30,891 (23.6)	26,790 (21.3)	19,956 (18.8)	17,270 (15.9)
Antihypertensives, n (%)	29,614 (67.3)	37,001 (68.4)	46,784 (69.4)	72,499 (69.4)	90,388 (69.7)	86,731 (69.0)	72,482 (67.9)	73,408 (67.0)
Systolic blood pressure, mean mmHg	139.2 ± 17.0	138.3 ± 16.6	137.5 ± 16.4	137.3 ± 16.4	136.9 ± 16.1	136.9 ± 16.2	136.6 ± 16.2	136.6 ± 16.3
Diastolic blood pressure, mean mmHg	78.1 ± 9.6	78.2 ± 9.5	78.2 ± 9.6	78.3 ± 9.5	78.2 ± 9.6	78.6 ± 9.7	78.7 ± 9.7	79.0 ± 9.8
Total cholesterol, mean mmol/l	5.4 ± 0.8	5.4 ± 0.8	5.4 ± 0.9	5.4 ± 0.9	5.4 ± 0.9	5.5 ± 0.9	5.5 ± 0.9	5.5 ± 0.9
LDL cholesterol, mean mmol/l	3.3 ± 0.7	3.3 ± 0.7	3.4 ± 0.7	3.4 ± 0.7	3.4 ± 0.7	3.4 ± 0.7	3.4 ± 0.7	3.4 ± 0.7
HDL cholesterol, mean mmol/l	1.3 ± 0.3	1.3 ± 0.3	1.3 ± 0.3	1.3 ± 0.4	1.3 ± 0.4	1.3 ± 0.4	1.3 ± 0.4	1.3 ± 0.4
Triglycerides, mean mmol/l	1.7 ± 0.8	1.8 ± 0.8	1.8 ± 0.8	1.7 ± 0.8	1.7 ± 0.9	1.8 ± 1.0	1.8 ± 1.0	1.8 ± 1.0
eGFR, mean ml/min/1.73m	83.2 ± 23.5	85.4 ± 24.1)	85.6 ± 25.2	85.2 ± 24.0	86.1 ± 24.9	86.5 ± 25.4	85.9 ± 25.6	85.5 ± 26.5
Microalbuminuria, n (%)	6,635 (18.5)	7,645 (17.6)	9,500 (18.4)	13,023 (17.9)	15,186 (17.8)	14,519 (17.4)	12,846 (17.6)	13,458 (17.7)
Macroalbuminuria, n (%)	2,614 (6.6)	2,816 (6.0)	3,775 (6.8)	4,419 (5.7)	4,735 (5.4)	4,348 (5.4)	3,544 (5.3)	3,644 (5.4)
BMI, mean kg/m^2	29.9 ± 5.3	30.0 ± 5.3	30.0 ± 5.4	30.2 ± 5.4	30.2 ± 5.5	30.2 ± 5.5	30.1 ± 5.5	30.1 ± 5.5
Physical activity < once a week[a], n (%)	7,230 (21.9)	8,068 (21.2)	10,778 (23.9)	14,408 (25.1)	16,240 (25.0)	51,036 (23.8)	15,783 (24.1)	16,854 (26.0)
Smoking[b], n (%)	5,893 (14.5)	7,393 (15.5)	9,056 (16.2)	15,073 (17.3)	18,792 (18.4)	17,544 (17.1)	14,615 (17.2)	15,508 (18.2)

Abbreviations: HbA1c, haemoglobin A1c; LDL, low-density lipoprotein; HDL, high-density lipoprotein; eGFR, estimated glomerular filtration rate; BMI, body mass index.

[a]30-minute walk or equivalent

[b]smoking at least one cigarette or pipe per day, or quit smoking within three months

[c]ATC code B01AC and N02BA01
