# Supplementary material for: Prescription of lipid-lowering medications for patients with type 2 diabetes mellitus and risk-associated LDL cholesterol: a nationwide study of guideline adherence from the Swedish National Diabetes Register
Source: BMC Health Serv Res. 2018 Nov 28;18:900. doi: 10.1186/s12913-018-3707-4 (PMC6260691; doi:10.1186/s12913-018-3707-4)
Supplement: Supplementary file 2 — Patient characteristics by year for observations attributed to secondary prevention. (PDF 29 kb) [file 12913_2018_3707_MOESM2_ESM.pdf]

Characteristics	**2007** n=24,405	**2008** n=31,013	**2009** n=40,178	**2010** n=63,712	**2011** n=78,469	**2012** n=75,393	**2013** n=65,156	**2014** n=69,097
Men, n (%)	15,096 (61.9)	19,152 (61.8)	24,736 (61.6)	38,758 (60.8)	47,763 (60.9)	46,266 (61.4)	40,280 (61.8)	42,840 (62.0)
Age, mean	70.8 ± 9.3	71.0 ± 9.3	71.1 ± 9.5	71.2 ± 9.6	71.4 ± 9.5	71.5 ± 9.5	71.7 ± 9.6	72.0 ± 9.5
Diabetes duration, mean years	10.1 ± 8.2	10.0 ± 8.2	10.2 ± 8.3	10.1 ± 8.2	10.3 ± 8.4	10.6 ± 8.5	11.0 ± 8.5	11.4 ± 8.6
HbA1c, mean mmol/mol	53.9 ± 12.4	54.5 ± 12.4	54.9 ± 12.8	55.4 ± 13.0	55.6 ± 13.6	56.2 ± 14.0	55.7 ± 14.4	55.9 ± 14.6
Diabetes medications, n (%)	18,947 (77.8)	23,824 (76.9)	30,857 (76.9)	49,906 (78.4)	61,573 (78.5)	59,776 (79.7)	51,438 (79.0)	54,066 (78.6)
Antiplatelets[c], n (%)	18,727 (77.9)	23,355 (76.7)	30,018 (76.4)	47,050 (75.1)	57,180 (74.0)	53,678 (72.4)	43,899 (68.9)	42,383 (63.4)
Antihypertensives, n (%)	22,087 (91.4)	28,113 (91.7)	36,693 (92.1)	58,076 (92.0)	70,801 (91.6)	67,506 (90.6)	57,568 (89.5)	61,091 (89.3)
Systolic blood pressure, mean mmHg	138.4 ± 17.7	137.5 ± 17.3	136.9 ± 17.1	136.4 ± 17.0	136.1 ± 16.8	135.6 ±16.7	135.6 ± 16.6	135.6 ± 16.7
Diastolic blood pressure, mean mmHg	75.0 ± 9.8	74.8 ± 9.8	74.8 ± 9.8	74.6 ± 9.8	74.7 ± 9.8	75.0 ± 9.8	75.0 ± 9.9	75.2 ± 9.9
Total cholesterol, mean mmol/l	4.8 ± 0.9	4.8 ± 0.9	4.8 ± 0.9	4.7 ± 0.9	4.8 ± 1.0	4.8 ± 1.0	4.7 ± 1.0	4.7 ± 1.0
LDL cholesterol, mean mmol/l	2.8 ± 0.7	2.7 ± 0.7	2.8 ± 0.8	2.8 ± 0.8	2.8 ± 0.8	2.7 ± 0.8	2.7 ± 0.8	2.7 ± 0.8
HDL cholesterol, mean mmol/l	1.2 ± 0.3	1.2 ± 0.3	1.2 ± 0.3	1.2 ± 0.3	1.2 ± 0.3	1.2 ± 0.4	1.2 ± 0.4	1.2 ± 0.3
Triglycerides, mean mmol/l	1.8 ± 0.8	1.8 ± 0.8	1.8 ± 0.8	1.8 ± 0.8	1.8 ± 0.9	1.8 ± 0.9	1.8 ± 1.0	1.8 ± 1.0
eGFR, mean ml/min/1.73m	73.3 ± 23.6	74.5 ± 23.9	74.3 ± 24.8	74.4 ± 24.3	74.7 ± 24.4	74.9 ± 25.6	74.2 ± 25.2	73.6 ± 26.6
Microalbuminuria, n (%)	4,741 (25.8)	5,661 (24.5)	7,324 (25.3)	11,320 (26.0)	13,047 (25.3)	12,457 (25.2)	11,237 (25.5)	11,911 (26.7)
Macroalbuminuria, n (%)	2,590 (12.1)	2,994 (11.5)	4,173 (12.9)	5,299 (11.2)	5,569 (10.4)	5,403 (11.2)	4,431 (10.9)	4,546 (11.6)
BMI, mean kg/m^2	29.4 ± 4.8	29.5 ± 4.9	29.6 ± 5.1	29.8 ± 5.1	29.8 ± 5.2	29.8 ±5.2	29.7 ± 5.2	29.6 ± 5.2
Physical activity < once a week[a], n (%)	5,989 (31.5)	6,887 (30.7)	9,117 (32.7)	12,035 (33.6)	13,532 (33.2)	13,305 (32.7)	13,170 (32.7)	13,451 (33.7)
Smoking[b], n (%)	2,630 (11.9)	3,290 (12.2)	4,196 (12.8)	7,373 (14.1)	9,048 (14.6)	8,693 (14.1)	7,116 (13.7)	7,420 (14.2)

Abbreviations: HbA1c, haemoglobin A1c; LDL, low-density lipoprotein; HDL, high-density lipoprotein; eGFR, estimated glomerular filtration rate; BMI, body mass index.

[a]30-minute walk or equivalent

[b]smoking at least one cigarette or pipe per day, or quit smoking within three months

[c]ATC code B01AC and N02BA01
