# Supplementary material for: Prescription of lipid-lowering medications for patients with type 2 diabetes mellitus and risk-associated LDL cholesterol: a nationwide study of guideline adherence from the Swedish National Diabetes Register
Source: BMC Health Serv Res. 2018 Nov 28;18:900. doi: 10.1186/s12913-018-3707-4 (PMC6260691; doi:10.1186/s12913-018-3707-4)
Supplement: Supplementary file 4 — Probability of prescribing lipid-lowering medications by year for patients younger than 80. (PDF 61 kb) [file 12913_2018_3707_MOESM4_ESM.pdf]

	Primary prevention		Secondary prevention	
	Crude n=692,343	Adjusted* n=220,094	Crude n=352,793	Adjusted* n=115,630
Year	Mean (95% CI)	Mean (95% CI)	Mean (95% CI)	Mean (95% CI)
2007	35.2 (34.7–35.6)	42.2 (40.7–43.6)	74.0 (73.4–74.5)	64.9 (62.9–66.9)
2008	39.6 (39.2–40.0)	46.2 (44.8–47.6)	76.2 (75.8–76.7)	68.6 (66.8–70.4)
2009	43.5 (43.1–43.8)	49.8 (48.4–51.2)	78.0 (77.6–78.4)	71.4 (69.7–73.0)
2010	45.6 (45.3–45.9)	52.4 (51.0–53.7)	78.2 (77.8–78.5)	72.9 (71.3–74.6)
2011	47.8 (47.5–48.1)	52.6 (51.2–54.0)	78.6 (78.3–79.0)	73.8 (72.1–75.4)
2012	48.7 (48.4–49.0)	52.6 (51.2–54.0)	77.4 (77.1–77.8)	72.2 (70.4–73.8)
2013	49.1 (48.8–49.4)	52.6 (51.1–54.0)	76.1 (75.7–76.5)	70.7 (68.9–72.5)
2014	50.1 (49.7–50.4)	52.0 (50.6–53.5)	75.5 (75.1–75.9)	70.4 (68.6–72.1)

* Adjusted for year, county council, type of care, sex, age, HbA1c, eGFR, diabetes duration, diabetes medications, antihypertensives, antiplatelets, blood pressure, microalbuminuria, macroalbuminuria, BMI, physical activity, smoking and cholesterol levels.
