# Supplementary material for: Prescription of lipid-lowering medications for patients with type 2 diabetes mellitus and risk-associated LDL cholesterol: a nationwide study of guideline adherence from the Swedish National Diabetes Register
Source: BMC Health Serv Res. 2018 Nov 28;18:900. doi: 10.1186/s12913-018-3707-4 (PMC6260691; doi:10.1186/s12913-018-3707-4)
Supplement: Supplementary file 5 — Probability of prescribing lipid-lowering medications by year for patients with eGFR > 30 ml/min/1.73 m2. (PDF 61 kb) [file 12913_2018_3707_MOESM5_ESM.pdf]

	Primary prevention		Secondary prevention	
	Crude n=751,864	Adjusted* n=237,751	Crude n=437,618	Adjusted* n=140,513
Year	Mean (95% CI)	Mean (95% CI)	Mean (95% CI)	Mean (95% CI)
2007	34.6 (34.2–35.0)	36.1 (34.7–37.5)	70.7 (70.2–71.2)	58.7 (56.8–60.6)
2008	38.9 (38.5–39.3)	40.2 (38.9–41.6)	72.8 (72.4–73.3)	63.2 (61.4–64.9)
2009	42.7 (42.3–43.0)	43.7 (42.4–45.1)	74.7 (74.3–75.1)	66.3 (64.6–68.0)
2010	44.7 (44.4–45.0)	46.3 (44.9–47.7)	74.8 (74.5–75.1)	68.0 (66.4–69.7)
2011	46.7 (46.4–47.0)	46.6 (45.2–48.0)	75.2 (74.9–75.5)	69.3 (67.7–70.9)
2012	47.4 (47.1–47.7)	46.6 (45.2–48.0)	73.7 (73.3–74.0)	68.1 (66.4–69.7)
2013	47.6 (47.3–47.9)	46.6 (45.2–48.0)	72.0 (71.6–72.3)	67.1 (65.4–68.8)
2014	48.3 (48.0–48.7)	46.1 (44.7–47.5)	71.1 (70.7–71.5)	66.1 (64.4–67.8)

* Adjusted for year, county council, type of care, sex, age, HbA1c, eGFR, diabetes duration, diabetes medications, antihypertensives, antiplatelets, blood pressure, microalbuminuria, macroalbuminuria, BMI, physical activity, smoking and cholesterol levels.
